# Genetic Profiling Reveals Cross-Contamination and Misidentification of 6 Adenoid Cystic Carcinoma Cell Lines: ACC2, ACC3, ACCM, ACCNS, ACCS and CAC2

**DOI:** 10.1371/journal.pone.0006040

**Published:** 2009-06-25

**Authors:** Janyaporn Phuchareon, Yoshihito Ohta, Jonathan M. Woo, David W. Eisele, Osamu Tetsu

**Affiliations:** 1 Head and Neck Cancer Research Laboratory, Department of Otolaryngology-Head and Neck Surgery, School of Medicine, University of California San Francisco, San Francisco, California, United States of America; 2 Helen Diller Family Comprehensive Cancer Center, University of California San Francisco, San Francisco, California, United States of America; 3 Genomics Core Facility, Institute for Human Genetics, School of Medicine, University of California San Francisco, San Francisco, California, United States of America; Mizoram University, India

## Abstract

Adenoid cystic carcinoma (ACC) is the second most common malignant neoplasm of the salivary glands. Most patients survive more than 5 years after surgery and postoperative radiation therapy. The 10 year survival rate, however, drops to 40%, due to locoregional recurrences and distant metastases. Improving long-term survival in ACC requires the development of more effective systemic therapies based on a better understanding of the biologic behavior of ACC. Much preclinical research in this field involves the use of cultured cells and, to date, several ACC cell lines have been established. Authentication of these cell lines, however, has not been reported. We performed DNA fingerprint analysis on six ACC cell lines using short tandem repeat (STR) examinations and found that all six cell lines had been contaminated with other cells. ACC2, ACC3, and ACCM were determined to be cervical cancer cells (HeLa cells), whereas the ACCS cell line was composed of T24 urinary bladder cancer cells. ACCNS and CAC2 cells were contaminated with cells derived from non-human mammalian species: the cells labeled ACCNS were mouse cells and the CAC2 cells were rat cells. These observations suggest that future studies using ACC cell lines should include cell line authentication to avoid the use of contaminated or non-human cells.

## Introduction

Adenoid cystic carcinoma (ACC) is the second most common malignant neoplasm of the salivary glands [1]–[8]. It is composed of duct-type epithelial cells and myoepithelial cells and shows variable pathological patterns. ACC occurs most frequently in men and women in their fifties. This malignancy arises in the major and the minor salivary glands but is more common in the minor salivary glands. As the tumor grows, it has a tendency to invade nerves, resulting in pain, numbness, and/or paralysis. ACC grows slowly and regional lymph node metastases are uncommon. Most patients with ACC survive more than 5 years after surgery and postoperative radiation therapy. Nevertheless, the survival rate at 10 years drops to 40% due to locoregional recurrences and distant metastases. Metastasis occurs most commonly in the lungs and less commonly in the liver, brain and bone [3], [4], [8]. Distant metastases can develop despite locoregional tumor control and can occur more than ten years after initial therapy. Due to this behavior, ACC is considered by some to be a systemic disease with an unpredictable clinical course [7], [8].

The best survival rates for ACC are gained by using a combination therapy involving surgery and postoperative radiation therapy [8]. Conventional chemotherapy has a poorly defined role in the treatment of ACC. Improved systemic therapies are clearly needed for ACC, and one important way to gain insight is to better understand the biological behavior of ACC cells.

Cell lines are frequently used to identify diagnostic biomarkers and for early studies of therapeutic development. To date, approximately ten ACC cell lines including ACC2, ACC3, ACCM, ACCS, ACCNS, and CAC2 have been established [9]–[16]. The ACC cell lines are not housed in Biological Resources Centers (BRCs). Rather, they have been exchanged between laboratories.

Despite their wide use in academic research, authentication of the established ACC cell lines has not been performed. Very recently, Choi et al reported that the ACC2, ACC3 and ACCM had identical genotypes. On comparison to the genotypes of the ATCC (American Type Culture Collection) cancer cell line collection, they found that the genotype of the cells they tested was identical to that of HeLa cells [17].

It is not yet clear whether alternative authenticated genuine ACC cell lines are available among the rest of the ACC cell lines. We used DNA fingerprint analysis short tandem repeat (STR) profiling to authenticate the six ACC cell lines cited above, which include ACC2, ACC3 and ACCM. STR profiling is currently accepted as an international reference standard for human cell line authentication [18]. Approximately 700 out of 1700 tumor cell lines at ATCC are STR profiled (http://www.atcc.org/CulturesandProducts/CellBiology/STRProfileDatabase/tabid/174/Default.aspx).

STR profiling techniques were originally developed for forensic applications [18]. The technology allows easy determination of a cell line's authenticity at minimal cost. We used the same system that the ATCC and JCRB (Japanese Collection of Research Bioresources) use for creating their databases (Promega's PowerPlex 1.2 system). This system covers eight STR loci. Each locus consists of short repetitive sequence elements 4 to 5 base pairs in length. These repeats are well-distributed throughout the human genome and are a rich source of highly polymorphic markers, which can be detected using the polymerase chain reaction (PCR). Alleles of STR loci are differentiated by the number of copies of the repeat sequence and are distinguished from one another using fluorescence detection following electrophoretic separation. The result is as a simple numerical code corresponding to the length of the PCR products amplified at each locus. This code enables identification of individuals with unprecedented accuracy. In combination with the amelogenin (AMEL) sex-typing gene, complete matching probabilities of nine STR loci are less than one in ten billion.

## Results

### Genetic profiling reveals that ACC2, ACC3 and ACCM were contaminated with HeLa cells

We performed DNA fingerprint analysis of ACC2, ACC3, and ACCM through STR examinations. We tested two different batches of ACC2 from two unrelated laboratories (ACC2/Sa and ACC2/Zh). Table 1 shows STR profiling for ACC2/Sa, ACC2/Zh, ACC3, and ACCM cells. We also tested 12 human cell lines at our institution, including HeLa cervical carcinoma cells. The repeat numbers for the HeLa (Table 1) and other 11 cells (data not shown) were perfectly or almost perfectly matched to their numbers in the ATCC database. This result also confirmed that contamination did not occur when we used these cells in our laboratory during the last twelve years.

**Table 1:** STR DNA fingerprint analysis of HeLa, ACC2, ACC3, and ACCM cells.

STR Locus										
Cell line	AMEL	CSF1PO	D13S317	D16S539	D5S818	D7S820	TH01	TPOX	vWA	Percent match to HeLa
HeLa	X	9, 10	12, 13.3	9, 10	11, 12	8, 12	7	8, 12	16, 18	100
ACC2/Sa	X	9, 10	12, 13.3	9, 10	11, 12	8, 12	7	8, 12	16,17, 18	97
ACC2/Zh	X	9, 10	12, 13.3	9, 10	11, 12	8, 12	7	8, 12	16, 18	100
ACC3	X	9, 10	12, 13.3	9, 10	11, 12	8, 12	7	8, 12	17, 18	94
ACCM	X	9, 10	12, 13.3	9, 10	11, 12	8, 12	7	8, 12	17, 18	94

The table shows repeat numbers of allelic ladder components of eight STR loci and AMEL. Electrophoretic profiles of vWA and other markers for these cells are shown in Figures 1 and S2, S3, S4, S5, S6, S7, S8, S9, S10.

**Figure 1:**
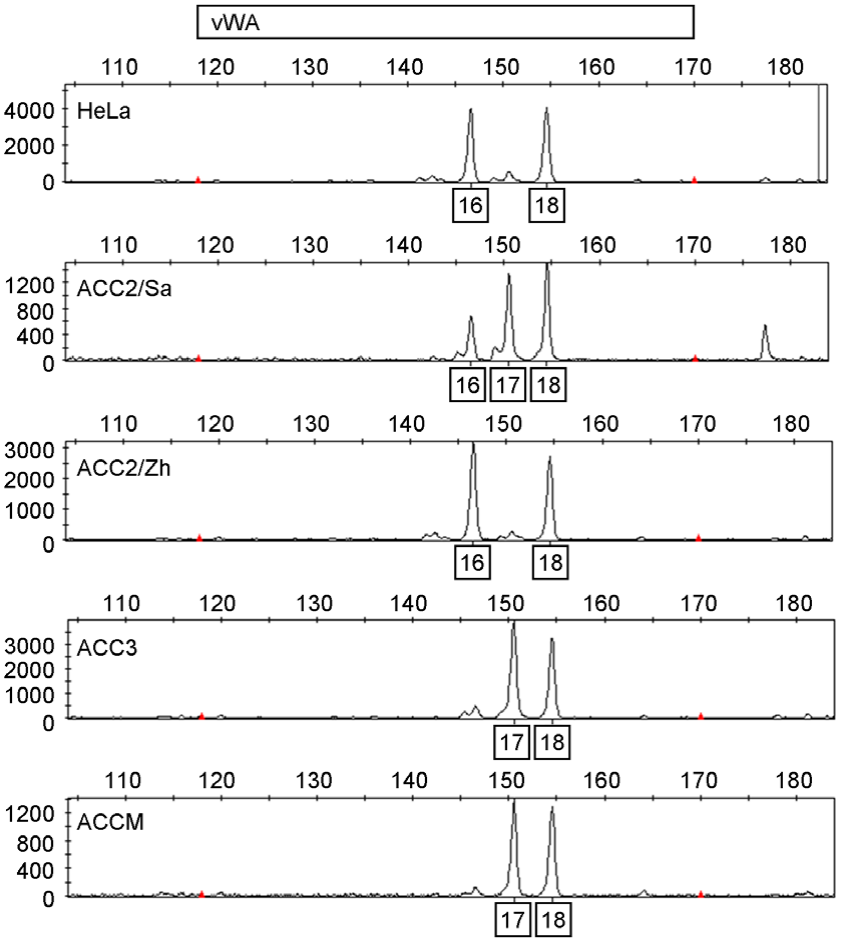
HeLa contaiminated-ACC2, ACC3, and ACCM demonstrate a variation in the von Willebrand factor gene (vWA). Electrophoretic profiles of the vWA marker for HeLa, ACC2/Sa, ACC2/Zh, ACC3, and ACCM are shown. Complete STR profiling is shown in Table 1 and Figures S2, S3, S4, S5, S6, S7, S8, S9, S10. A multiplex PCR reaction was performed using two-color detection fluorescent dye-linked primers. One ng of each genomic DNA was independently amplified in a 25 µl reaction volume. The amplified PCR products were separated by capillary electrophoresis on a 3730xI DNA Analyzer (Applied Biosystems) and analyzed using GeneMapper v4.0 software (Applied Biosystems). Cell lines for the study were generously provided by Drs. Takashi Saku (ACC2/Sa and ACC3), Naishuo Zhu (ACC2/Zh), Kanemitsu Shirasuna (ACCS), Noriaki Tanaka (ACCM and ACCNS), and Ruy Jaeger (CAC2). HeLa cells were purchased from the ATCC.

The ACC2 cell line was derived from a 28-year-old female patient and ACC3 came from a 49-year-old male [9]. ACCM was established from ACC2 as a highly metastatic subclone [10]. Our STR analysis demonstrated that our ACC2, ACC3, and ACCM cells had nearly identical electrophoretic profiles (Table 1, and Figures 1 and S1, S2, S3, S4, S5, S6, S7, S8, S9, S10). A very similar observation was reported from another laboratory [17]. Amelogenin (AMEL) gender-typing showed that all three cell lines lacked a Y chromosome. This observation suggested that all three cell lines were derived from a female patient. It is, however, important to note that the Y chromosome is often lost in cultured cells. Therefore it use as a gender discrimination tool is not always accurate in cultured cells [19], [20]. We found a small variation in the von Willebrand factor gene (vWA) that even occurred between the two ACC2 lines (Figure 1). This finding was confirmed by a different set of STR analyses (Figure S1).

Surprisingly, the STR profiles of these three cell lines were almost identical to that of HeLa cells, except for vWA in ACC2/Sa, ACC3 and ACCM (Table 1, and Figures 1 and S1). We purchased HeLa cells from the ATCC and found that our HeLa cells had the same STR profile as the ATCC HeLa cells. HeLa are used extensively around the world, and are known as aggressive contaminators of other cells [21]. There is no common stock of HeLa cells, and each batch from each source is slightly different [21]. Studies have demonstrated that HeLa cells, their subline HeLaS3, and their cross-contaminants (Hep2, Intestine 407, KB, and Chang Liver) are closely related by STR profiling, but are not necessarily identical. In fact, there is even a slight discrepancy between three HeLaS3 subclones (CCL2.2, IFO50011 and JCRB9010) in the JCRB database in D13S317, D5S818 and vWA STR profiling (Table S1). We found that our ACC cells had a small variation in the vWA locus: (16, 18), (17, 18), and (16, 17, 18). An identical vWA profiling change was found in the HeLaS3 subclones (Table S1). Thus, we attributed differences between them to genetic instability and variations in cultivating conditions [21], [22].

Various studies have determined that cross-contaminated cells are defined as those that match at 80% or more of alleles in STR analysis [22], [23]. The percent matches of our three ACC cell lines to HeLa cells were 100%, 97% or 94% (Table 1). These observations suggest that the original ACC2, ACC3, and ACCM cells had been replaced with HeLa cells. ACC2, ACC3 and ACCM were first reported in 1986 and 1997, many years after the establishment of HeLa cells in 1951 [21], [23]. Therefore, it is possible that ACC2, ACC3 and ACCM may have acquired the genetic changes particularly in vWA due to passaging effects in HeLa cells [24].

Taken together, this data suggests that ACC2, ACC3, and ACCM were actually HeLa cells.

### ACCS was genetically identical to T24 urinary bladder cancer cells

We also performed STR analysis on ACCS, another ACC cell line (Table 2, and Figures S11 and S12). Because the ACCS line's genetic profile did not resemble those of other cell lines we tested in this study (Table 1 and data not shown), we compared our results with information in the ATCC and JCRB databases (Table 2, http://cellbank.nibio.go.jp/str2/str006.html).

**Table 2:** STR DNA fingerprint analysis of ACCS.

STR Locus										
Cell line	AMEL	CSF1PO	D13S317	D16S539	D5S818	D7S820	TH01	TPOX	vWA	Percent match to ACCS
ACCS	X	12	12	9	10	10, 11	6	8	17	100
ECV304	X	12	12	9	10	10, 11	6	8, 11	17	95
T24	X	10, 12	12	9	10, 12	10, 11	6	8, 11	17	87
EJ-1	X	10, 12	12	9	10, 12	10, 11	6	8, 11	17	87

STR profiling of ACCS cells was compared to data in the Japanese Collection of Research Bioresources (JCRB) database (ECV304, T24 and EJ-1 cells). Electrophoretic profiles of the complete STR markers for ACCS cells are shown in Figures S11 and S12.

To our surprise, its profile was nearly identical to that of T24 urinary bladder cancer cells and their contaminants, EJ-1 and ECV304 cells (Table 2). The percent matches of the STR profiles of these cells to ACCS were 95% and 87%, suggesting that there was cross-contamination of ACCS with T24 cells [22], [25]. T24 is a common cross-contaminating cell line [22], [26]. The expression of HLA antigen on the cell surface revealed that EJ-1 cells share the same genetic profile with the original T24 bladder cancer cells [26]. ECV304 was initially reported as an endothelial cell line derived from spontaneously transformed umbilical vein [27]. However, it was subsequently determined to be a contaminant of T24 [22], [25], [28]–[30]. T24 is a chromosomally unstable cell line and acquires secondary genetic changes [22], [31]. This fact explains why the STR result for ECV304 was not identical to that of T24 (Table 2). ECV304 lost one allele of D5S818 (5p21–q31) and another allele of CSF1PO (5q33.3–q34) from the original T24 cells [25]. Interestingly, these deletions were also found in the ACCS cells, suggesting that D5S818 and CSF1PO are deletion-prone loci in T24 cells. In addition, the ACCS cells had a further allelic deletion in TPOX (2p23-2pter). ACCS and ECV304 were first reported in 1990, many years after T24 cells were established in 1970 [27], [32]. Therefore, it is possible that ACCS and ECV304 may have acquired their genetic changes due to passaging effects in T24 cells [24].

Overall, we concluded that the ACCS cells were genetically identical to T24 urinary bladder transitional cell carcinoma cells.

### ACCNS cells originated from a mouse cell line and CAC2 cells were from a rat cell line

We were unable to amplify any human polymorphic STR markers in our ACCNS and CAC2 cells (data not shown). This result suggested that these cells were not human cells. Cross-contamination among cell lines is not limited to intraspecies contamination, and interspecies contamination can occur. To test if ACCNS and CAC2 cells were non-human, we performed a cytochrome C oxidase subunit I (COI) DNA barcoding assay for species identification [33]. This is a multiplex PCR-base assay to detect cross-contaminations among the most common culture species. The assay uses COI, a conserved mitochondrial gene, whose use is a global standard for species identification [34]. The 5′ end of the COI region, which is 648 nucleotide base pairs long in most groups of higher animals, contains substantial interspecies variation— yet intraspecies variation remains relatively low in nearly all animals [34]. This approach enables the identification of a wide array of animals with high confidence. This technique is currently applied to species identification in cell cultures [33], [35].

**Figure 2:**
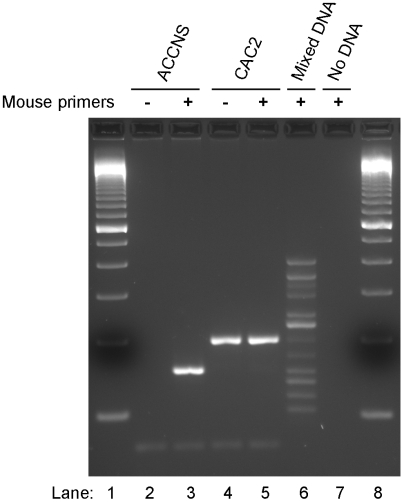
ACCNS cells were mouse cells and CAC2 cells were rat cells. A cytochrome C oxidase subunit I (COI) DNA barcoding assay was performed using multiplex species-specific primer sets for 12 species (+, lanes 3, 5, 6 and 7) or 11 species-specific sets without mouse primers (−, lanes 2 and 4). The PCR products were separated in a 2% agarose gel and visualized with ethidium bromide following amplification of 5′ end of COI region. Lanes 1 and 8: 100 bp ladders. Lanes 2 and 3: ACCNS. Lanes 4 and 5: CAC2. Lane 6: Mixed DNA template from 12 species. Lane 7: no template DNA. The animals and expected PCR product sizes were as follows; human (391 bp), cat (341 bp), Chinese hamster (315 bp), rhesus monkey (287 bp), horse (243 bp), African green monkey (222 bp), rat (196 bp), dog (172 bp), mouse (150 bp), rabbit (136 bp), goat (117 bp), and cow (102 bp). The PCR products were sequenced to confirm that they contained the species-specific nucleotide sequence.

We isolated genomic DNA from ACCNS and CAC2 cells and amplified it by PCR using multiplex 12 species-specific primer sets designed to amplify a specifically sized product only in the presence of target species [33]. We next separated the PCR products in a 2% agarose gel and visualized them with ethidium bromide (Figure 2). We used two sets of PCR primers. Lanes 3 and 5 included all 12 species-specific PCR primer sets, whereas we eliminated mouse primer pairs in lanes 2 and 4. We used the mouse-free PCR primer sets because they would not compete with the human primer pair if the human genes were present in very low copy levels. Lanes 2 and 3 show results from ACCNS cells and lanes 4 and 5 show results for CAC2 cells. Lane 6 is a positive control and lane 7 is a negative control. We found a 150 bp PCR product in lane 3. This PCR product did not appear when the mouse primer pairs were absent (lane 2). These observations suggested that ACCNS cells were a mouse cell line. On the other hand, lanes 4 and 5 showed 196 bp PCR products, indicating that CAC2 cells were derived from rats. The PCR products were further analyzed by sequencing, and we confirmed that ACCNS cells were mouse cells and that CAC2 cells were rat cells (data not shown).

## Discussion

We investigated six established and widely-used ACC cell lines and found that all six had been replaced with other cells (Table 3). The cells labeled ACC2, ACC3, and ACCM were HeLa cells, while the ACCS cells were human bladder cancer cells T24 (intraspecies cross-contamination). The putative ACCNS and CAC2 cells were actually derived from mice (ACCNS) or rats (CAC2; interspecies cross-contamination). Our ACC cells were never cultured simultaneously with HeLa cells, and no T24, rat or mouse cells were cultured in our incubators. Thus, we did not cause the contamination. None of these cells were derived from ACC patients. These results unfortunately call into question the validity of results from prior studies using these cell lines.

**Table 3:** Misidentified and cross-contaminated ACC cell lines.

ACC cell line	Real identity	Actual human malignancy or Species	Evidence
ACC2	HeLa	Uterine adenocarcinoma	STR
ACC3	HeLa	Uterine adenocarcinoma	STR
ACCM	HeLa	Uterine adenocarcinoma	STR
ACCS	T24	Urinary bladder carcinoma	STR
ACCNS	?	Mouse cells	DNA barcoding
CAC2	?	Rat cells	DNA barcoding

Cell lines established from human tissues are extensively used to study human diseases and other conditions. Lines derived from malignant neoplasms are particularly useful tools for investigating mechanisms of tumor initiation and development. These cells are often used as pre-clinical models for the disease, as sources for biomarker or drug target identification, and as vessels to screen for efficacy hits and for early toxicity indicators. Because of the increasing importance of cultured cells in cancer research and other biomedical studies, cross-contamination of cell lines is becoming a more frequently encountered problem. It has been reported that approximately 20% of cell lines are incorrectly designated [21], [36]–[38]. Nevertheless, researchers infrequently authenticate cell lines prior to initiating studies or before freezing stocks. The NIH has recently recognized the problem and has issued a notice that cell line authentication must accompany all grant applications and all publications of research findings (http://grants.nih.gov/grants/guide/notice-files/NOT-OD-08-017.html).

HeLa cells were the first established human cancer cell line [23]. They were quickly distributed worldwide with attendant contamination problems. Cross-contamination of cell lines with HeLa cells was first reported in 1968 [39]. Among the first established 20 human cell lines, 18 were eventually identified as HeLa cells [21], [39], [40]. In the past, various methods were developed to identify intraspecies contamination of cells in culture. Electrophoretic polymorphisms of glucose-6-phosphate dehydrogenase (G6PD) initially played a significant role in identifying HeLa contamination [41]. Phosphoglucomutase (PGM) electrophoretic polymorphisms also supported HeLa cell identification [41]. HeLa cells show Type A G6PD and Type1 PGM profiling. Besides these biochemical methods, a genetic approach has also been applied: HeLa cells display characteristic trypsin-Giemsa stained chromosome band patterns [41]. In addition, the presence or absence of a Y chromosome can be assessed by fluorescent staining. The expression of HLA antigen on the cell surface was an alternative approach to verify cell identification. This approach revealed that EJ-1 cells share the same genetic profile with T24 bladder cancer cells [26]. Currently STR profiling of polymorphic markers has replaced these methods. It is an efficient and reliable method for detecting cross-contamination of human cell lines. An earlier usage of STR showed that ECV304 was a contaminant of T24 cells [22], [25], [28].

Cell line contamination is a significant problem. For example, one study analyzed 550 leukemia-lymphoma cell lines and reported that 82 (14.9%) were cross-contaminated with different cells; some were redundant but others were not of leukemia-lymphoma origin [42]. Another study reported that the MDA-MB-435 human breast cancer cell line was thoroughly contaminated with the M14 human melanoma cell line [43]. M14 cross-contaminants have also been found in the thyroid cancer cell line NPA87 and its sublines, which make it the most frequent contaminant group in thyroid cancer cells, followed by HT-29 colon cancer contaminants [44].

Despite these facts, there is continued use of the above-mentioned cell lines, as reported in recent publications [42], [45], [46]. As a consequence, reports with invalid hypotheses and incorrect results have been published. Thus, use of contaminated cell lines may cause significant delays in the development of new treatments or new biomarkers. They may also add significant costs to the process if researchers spend time and money investigating false leads.

ACC studies are no exception to this problem [17]. We performed a PubMed search and found that 75 original research papers using the cell lines examined in this study were published from 1991 to 2008 (Table 4). Notably, ACC2 and ACCM were used in more than 30 published studies. Many of these reports proposed new therapeutic applications based on results derived from use of the cell lines. Our study suggests that ACC cell lines should be authenticated before research involving their use is performed.

**Table 4:** Number of articles citing 6 ACC cell lines.

Cell line	Number of articles
ACC2	30
ACC3	14
ACCM	32
ACCS	7
ACCNS	1
CAC2	10
Total	94

A PubMed search revealed that 75 original research papers citing at least one of the six cell lines analyzed here were published from 1991 to 2008. Some papers used more than one cell line and, thus, the total number was more than 75 publications.

If the contamination identified here is widespread among other cell lines that are identified as ACC cells, it will be necessary to establish new ACC cell lines. It will be essential to ensure that the new cell lines do not become contaminated.

As a first, step prior to the creation of cell lines, researchers should freeze and store multiple samples of the original material and then, when a cell line is established, they should perform STR profiling to confirm that the cells are identical to the donor tissue. Sporadic ACC tumors often overexpress c-Kit, keratin, S-100 protein, and actin [8]. Detection of these ACC-specific markers is an alternative approach to quality control of ACC cells. It should be performed in all new ACC cell lines. After verification, cell line stocks should be deposited in one or more BRCs before information about them is published. The quality control procedures at BRCs may reduce contamination problems. Additionally, because ACC is composed of duct-type epithelial cells and myoepithelial cells, it may be prudent to establish each component separately to avoid one of the cellular components becoming dominant during passaging.

The genotypes, karyotypes and phenotypes of new cell lines must be documented by the originators and/or the BRCs using cells with low passage numbers. This approach will avoid the effects of extended passage number on selective pressure and genetic instability in cell lines [24]. Researchers should also include data concerning STR profiling of donor tissue and the cell line in publications of studies that use the new cell line. An additional important measure would be to ensure that all personnel working with cultured cells are given thorough instructions on how to avoid contamination. Finally, publications of any ACC research using ACC cell lines should include cell line authentication. We anticipate that these efforts will considerably reduce cell contamination and thus improve the quality of ACC research.

## Materials and Methods

### Cell lines and cell culture

Cell lines were obtained as follows: ACC2/Sa and ACC3 were generously provided by Dr. Takashi Saku, Niigata University, Japan. ACC2/Zh cells were kindly donated by Dr. Naishou Zhu, Fudan University, Shanghai, PR China. ACCM and ACCNS were courtesy of Dr. Noriaki Tanaka, Hyogo College of Medicine, Japan. ACCS was a generous gift from Dr Kanemitsu Shirasuna, Kyushu University, Fukuoka, Japan. CAC2 was a gift from Dr. Ruy Jaeger, University of São Paulo, Brazil. HeLa cells were purchased from the ATCC. All cell lines were maintained as monolayer cultures in RPMI1640 containing penicillin-streptomycin, nonessential amino acids, sodium pyruvate, L-glutamine and 10% Fetal Bovine Serum (PAA Laboratories Inc., New Bedford, MA). They were incubated in a humidified atmosphere with 5% CO_2_ at 37°C.

### Genomic DNA isolation and short tandem repeat (STR) DNA fingerprint analysis

Genomic DNA was isolated from cell lines in a clean environment using the Wizard SV Genomic DNA Purification system (Promega, Madison, WI). In this system, co-purified RNA was removed by RNase A. The purity of genomic DNA was validated by monitoring the OD_260 nm_/OD_280 nm_ absorbance ratio [47]. DNA whose ratio was between 1.87 and 1.97 was used for the further analysis. STR DNA fingerprint analysis was performed using the Powerplex 1.2 system (Promega). The following STR markers were tested: AMEL (Xp22.10-22.3 and Y), CSF1PO (5q33.3–34), D13S317 (13q22–q31), D16S539 (16q24-qter), D5S818 (5q21–q31), D7S820 (7q), TH01 (11p15.5), TPOX (2p23-2pter), and vWA (12p12-pter). A multiplex PCR reaction was performed using two-color detection fluorescent dye-linked primers according to the manufacturer's manual. We amplified 1 ng of template DNA in a 25 µl reaction volume. The amplified PCR products were separated by capillary electrophoresis on a 3730xI DNA Analyzer (Applied Biosystems) and analyzed using GeneMapper v4.0 software (Applied Biosystems). For comparison, percent match of STR profiling was calculated between two cell lines according to the following mathematical formula [15], [17]: Percent match (%) = 100×(number of alleles in reference and study samples)×2/(total number of alleles in reference+total number of alleles in study samples).

### Cytochrome C oxidase subunit I (COI) DNA barcoding assay

PCR reactions were performed using multiplex 12 species-specific primer sets that target the 5′ variable region of the COI gene [33]. The complete list of primer sequences has been published [33]. PCR products were visualized on 2% agarose gel stained with ethidium bromide. PCR products were sent for sequencing analysis to confirm results.

## Supporting Information

Table S1 (0.08 MB PPT)

Figure S1 Electrophoretic profiles of the vWA marker for HeLa, ACC2/Sa, ACC2/Zh, ACC3, and ACCM cells are shown. A separate STR analysis from Table 1 and Figure 1 was performed at the Fragment Analysis Facility, Johns Hopkins University.
(4.03 MB TIF)

Figure S2 Electrophoretic profiles of the AMEL marker for HeLa, ACC2/Sa, ACC2/Zh, ACC3, and ACCM cells shown in Table 1 are presented.
(1.88 MB TIF)

Figure S3 Electrophoretic profiles of the CSF1PO marker for HeLa, ACC2/Sa, ACC2/Zh, ACC3, and ACCM cells shown in Table 1 are presented.
(1.90 MB TIF)

Figure S4 Electrophoretic profiles of the D13S317 marker for HeLa, ACC2/Sa, ACC2/Zh, ACC3, and ACCM cells shown in Table 1 are presented.
(1.85 MB TIF)

Figure S5 Electrophoretic profiles of the D16S539 marker for HeLa, ACC2/Sa, ACC2/Zh, ACC3, and ACCM cells shown in Table 1 are presented.
(1.80 MB TIF)

Figure S6 Electrophoretic profiles of the D5S818 marker for HeLa, ACC2/Sa, ACC2/Zh, ACC3, and ACCM cells shown in Table 1 are presented.
(1.77 MB TIF)

Figure S7 Electrophoretic profiles of the D7S820 marker for HeLa, ACC2/Sa, ACC2/Zh, ACC3, and ACCM cells shown in Table 1 are presented.
(1.75 MB TIF)

Figure S8 Electrophoretic profiles of the TH01 marker for HeLa, ACC2/Sa, ACC2/Zh, ACC3, and ACCM cells shown in Table 1 are presented.
(1.82 MB TIF)

Figure S9 Electrophoretic profiles of the TPOX marker for HeLa, ACC2/Sa, ACC2/Zh, ACC3, and ACCM cells shown in Table 1 are presented.
(1.87 MB TIF)

Figure S10 Electrophoretic profiles of the vWA marker for HeLa, ACC2/Sa, ACC2/Zh, ACC3, and ACCM cells shown in Table 1 are presented.
(1.88 MB TIF)

Figure S11 Electrophoretic profiles of the AMEL, CSF1PO, D13S317, D16S539, D5S818, D7S820, and TH01markers for ACCS cells shown in Table 2 are presented.
(1.62 MB TIF)

Figure S12 Electrophoretic profiles of the TPOX and vWA markers for ACCS cells shown in Table 2 are presented.
(0.83 MB TIF)
