# Supplementary material for: Genetic Profiling Reveals Cross-Contamination and Misidentification of 6 Adenoid Cystic Carcinoma Cell Lines: ACC2, ACC3, ACCM, ACCNS, ACCS and CAC2
Source: PLoS One. 2009 Jun 25;4(6):e6040. doi: 10.1371/journal.pone.0006040 (PMC2698276; doi:10.1371/journal.pone.0006040)
Supplement: Table S1 — (0.08 MB PPT) [file pone.0006040.s001.ppt]

## Slide 1
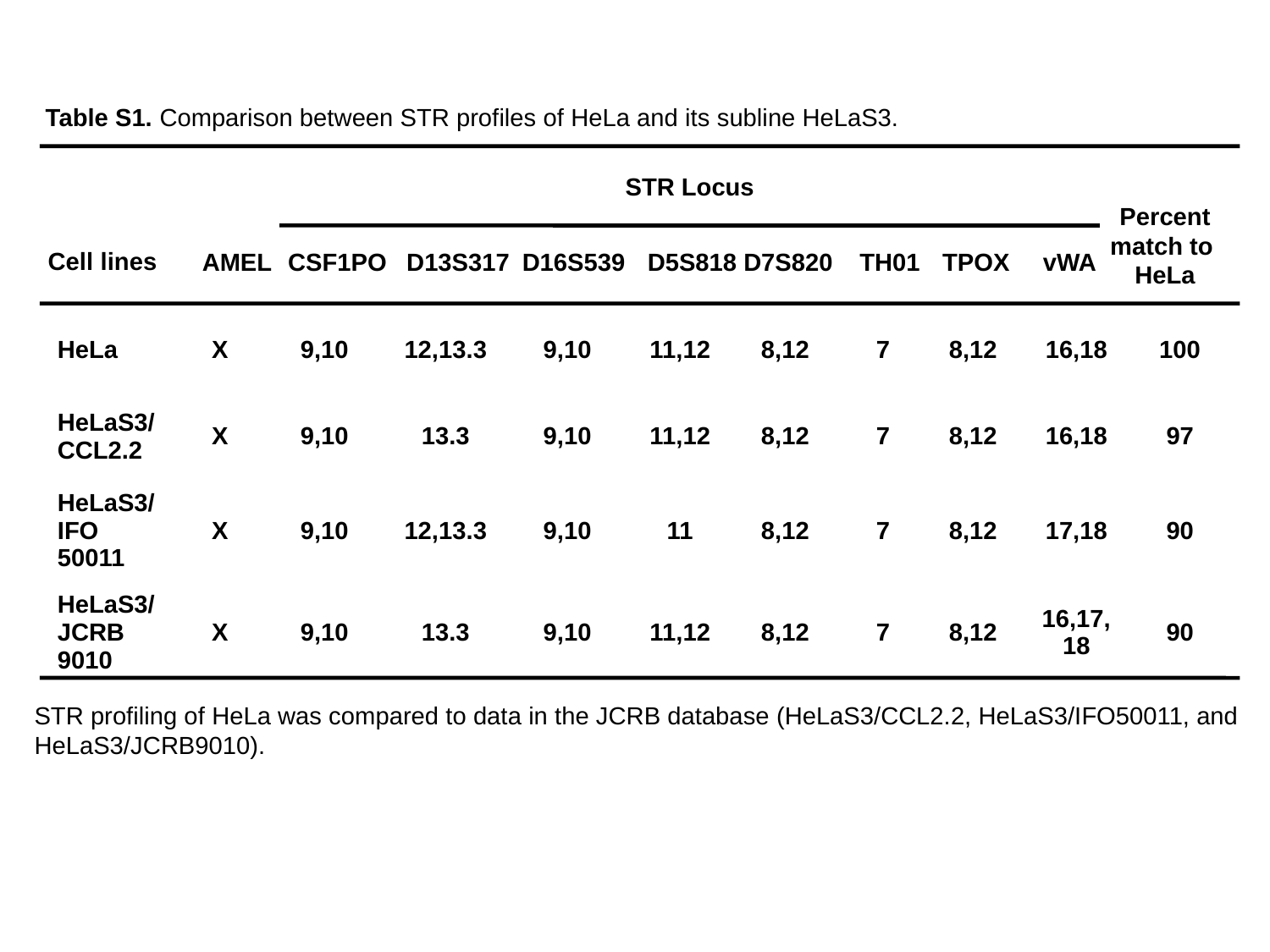

Table S1. Comparison between STR profiles of HeLa and its subline HeLaS3.
STR Locus
Percent
match to
HeLa
Cell lines
AMEL
CSF1PO
D13S317
D16S539
D5S818
D7S820
TH01
TPOX
vWA
| HeLa | X | 9,10 | 12,13.3 | 9,10 | 11,12 | 8,12 | 7 | 8,12 | 16,18 | 100 |
| --- | --- | --- | --- | --- | --- | --- | --- | --- | --- | --- |
| HeLaS3/ CCL2.2 | X | 9,10 | 13.3 | 9,10 | 11,12 | 8,12 | 7 | 8,12 | 16,18 | 97 |
| HeLaS3/ IFO 50011 | X | 9,10 | 12,13.3 | 9,10 | 11 | 8,12 | 7 | 8,12 | 17,18 | 90 |
| HeLaS3/ JCRB 9010 | X | 9,10 | 13.3 | 9,10 | 11,12 | 8,12 | 7 | 8,12 | 16,17, 18 | 90 |
STR profiling of HeLa was compared to data in the JCRB database (HeLaS3/CCL2.2, HeLaS3/IFO50011, and
HeLaS3/JCRB9010).
