# Supplementary figures and images for: Genetic Profiling Reveals Cross-Contamination and Misidentification of 6 Adenoid Cystic Carcinoma Cell Lines: ACC2, ACC3, ACCM, ACCNS, ACCS and CAC2
Source: PLoS One. 2009 Jun 25;4(6):e6040. doi: 10.1371/journal.pone.0006040 (PMC2698276; doi:10.1371/journal.pone.0006040)

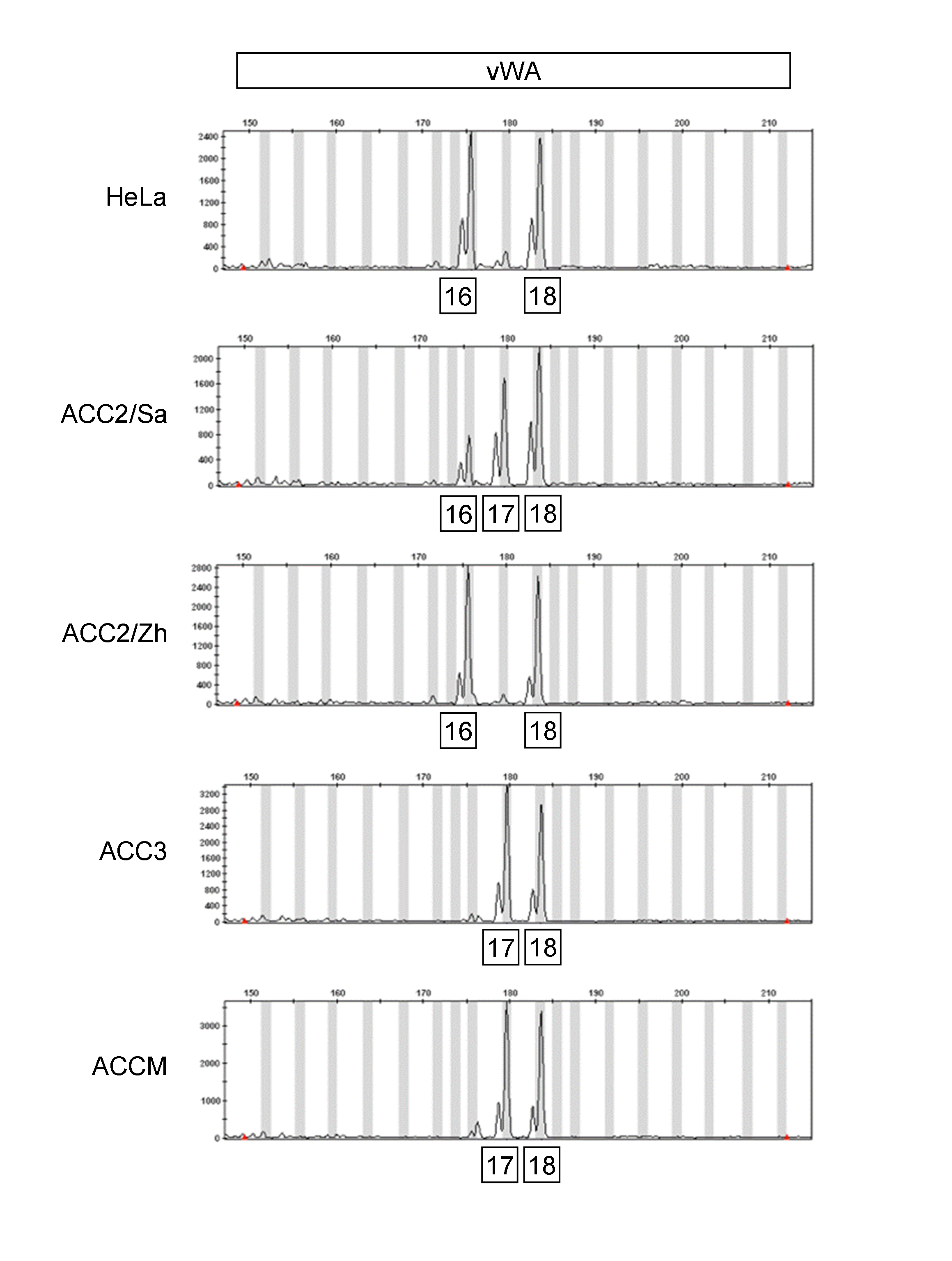

Supplement: Figure S1 — Electrophoretic profiles of the vWA marker for HeLa, ACC2/Sa, ACC2/Zh, ACC3, and ACCM cells are shown. A separate STR analysis from Table 1 and Figure 1 was performed at the Fragment Analysis Facility, Johns Hopkins University. (4.03 MB TIF) [file pone.0006040.s002.tif]

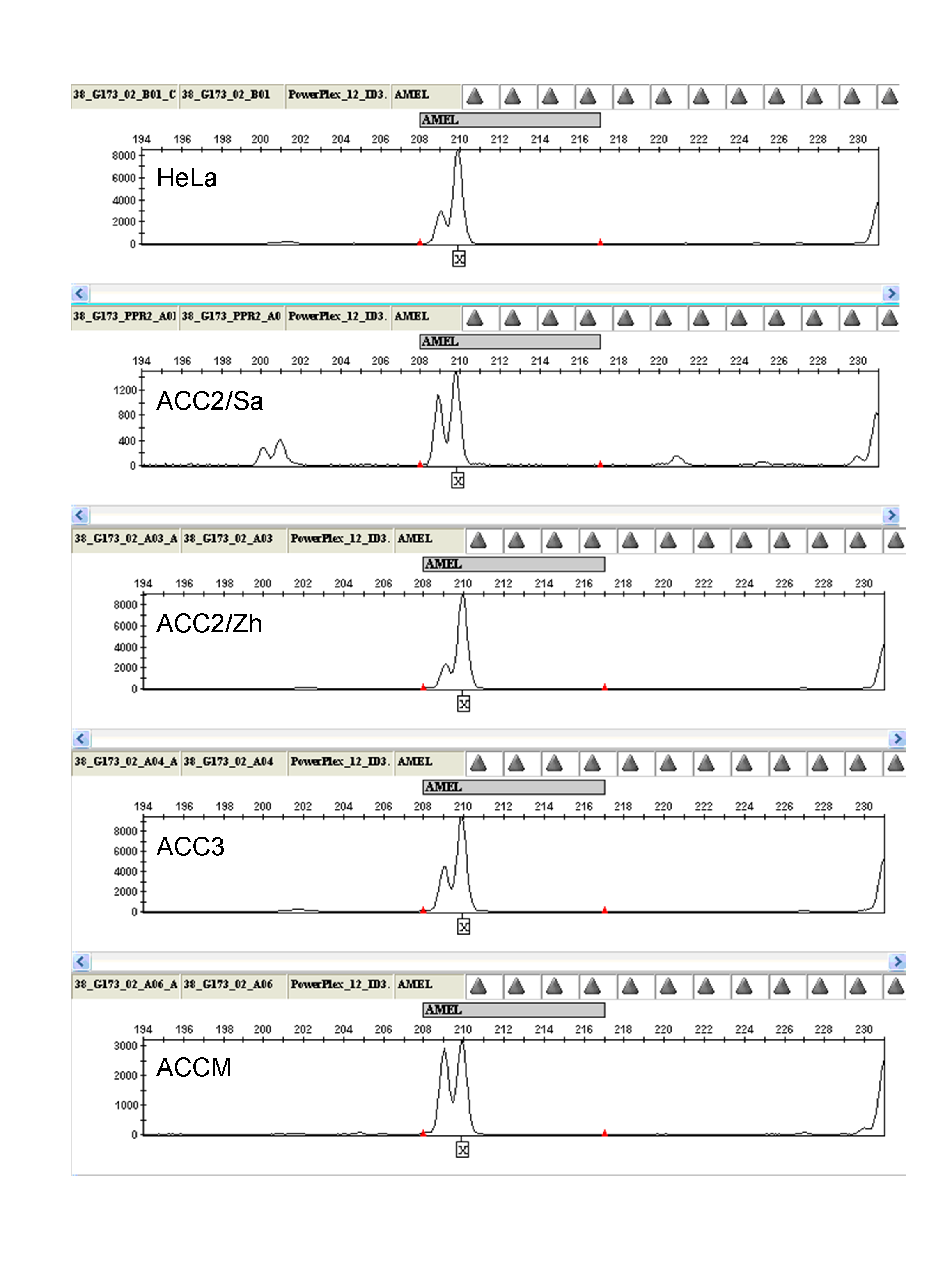

Supplement: Figure S2 — Electrophoretic profiles of the AMEL marker for HeLa, ACC2/Sa, ACC2/Zh, ACC3, and ACCM cells shown in Table 1 are presented. (1.88 MB TIF) [file pone.0006040.s003.tif]

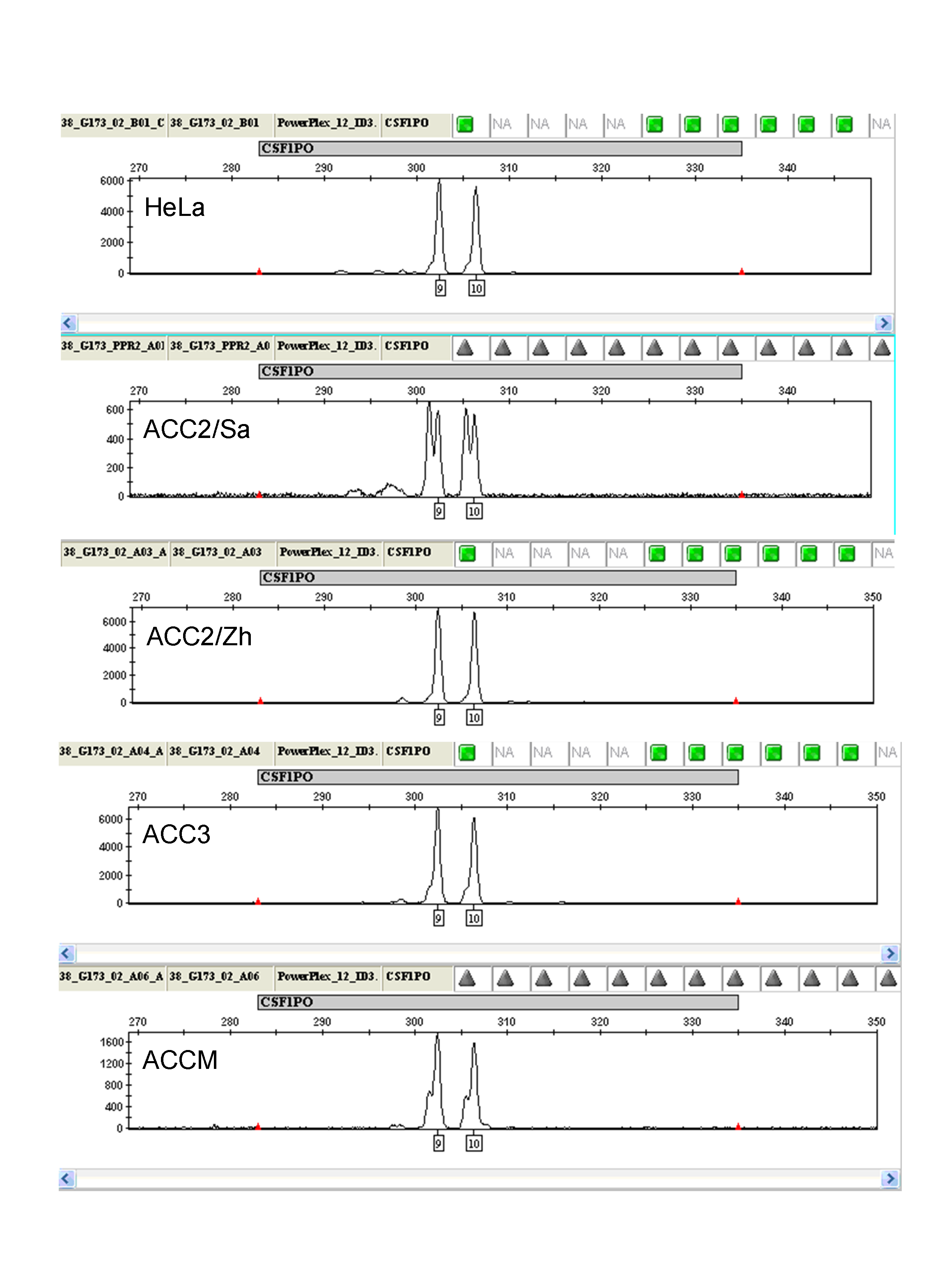

Supplement: Figure S3 — Electrophoretic profiles of the CSF1PO marker for HeLa, ACC2/Sa, ACC2/Zh, ACC3, and ACCM cells shown in Table 1 are presented. (1.90 MB TIF) [file pone.0006040.s004.tif]

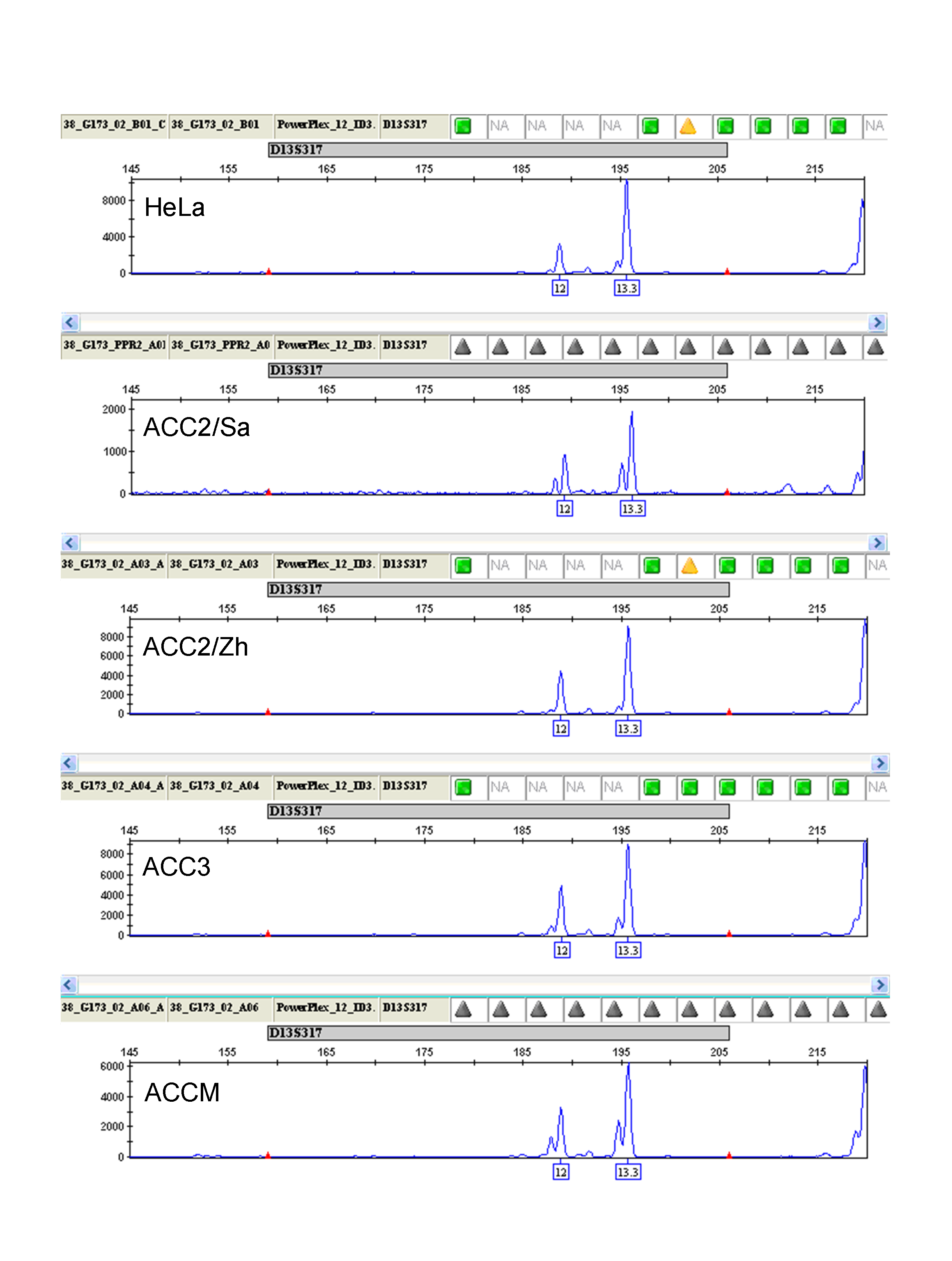

Supplement: Figure S4 — Electrophoretic profiles of the D13S317 marker for HeLa, ACC2/Sa, ACC2/Zh, ACC3, and ACCM cells shown in Table 1 are presented. (1.85 MB TIF) [file pone.0006040.s005.tif]

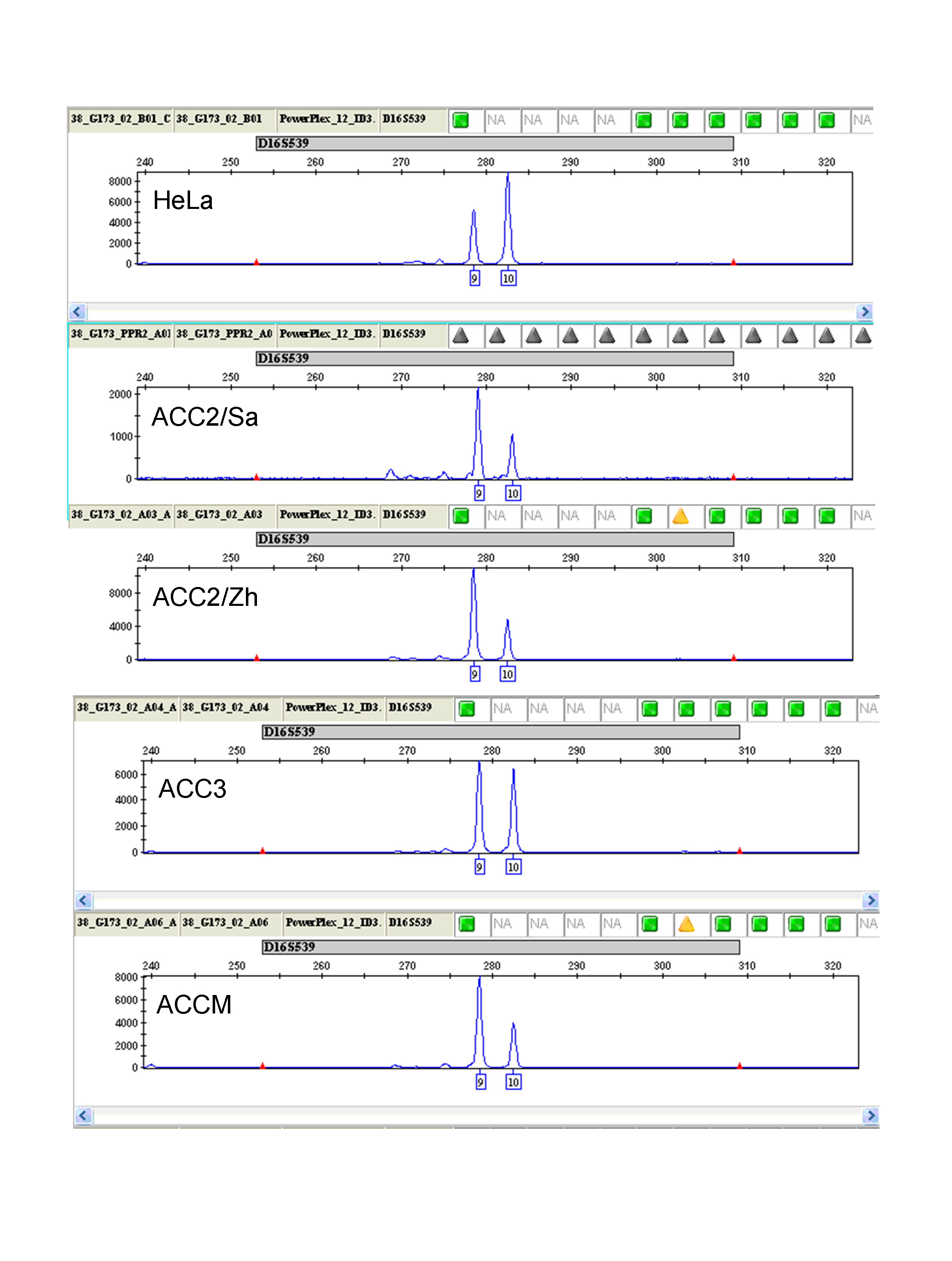

Supplement: Figure S5 — Electrophoretic profiles of the D16S539 marker for HeLa, ACC2/Sa, ACC2/Zh, ACC3, and ACCM cells shown in Table 1 are presented. (1.80 MB TIF) [file pone.0006040.s006.tif]

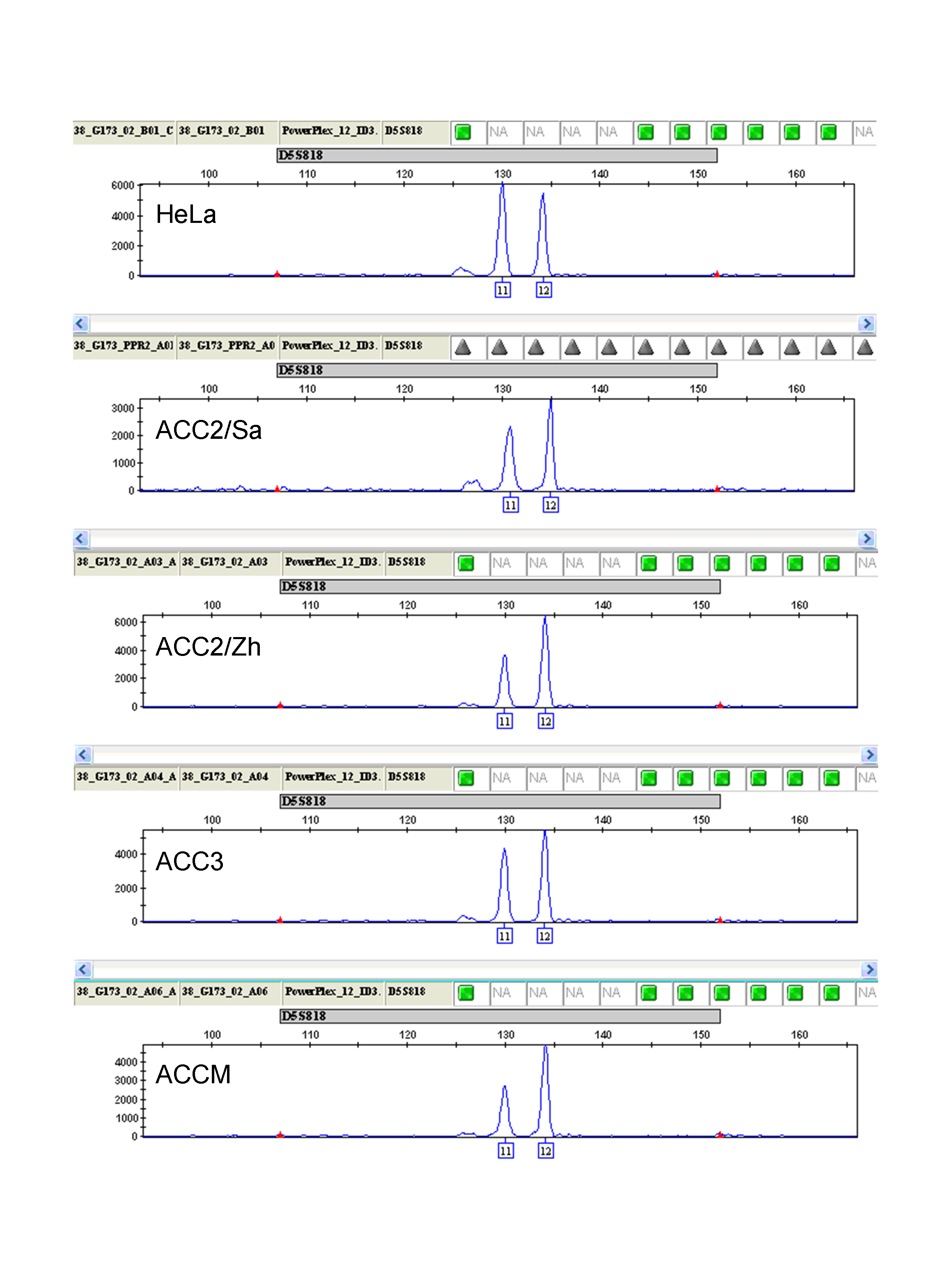

Supplement: Figure S6 — Electrophoretic profiles of the D5S818 marker for HeLa, ACC2/Sa, ACC2/Zh, ACC3, and ACCM cells shown in Table 1 are presented. (1.77 MB TIF) [file pone.0006040.s007.tif]

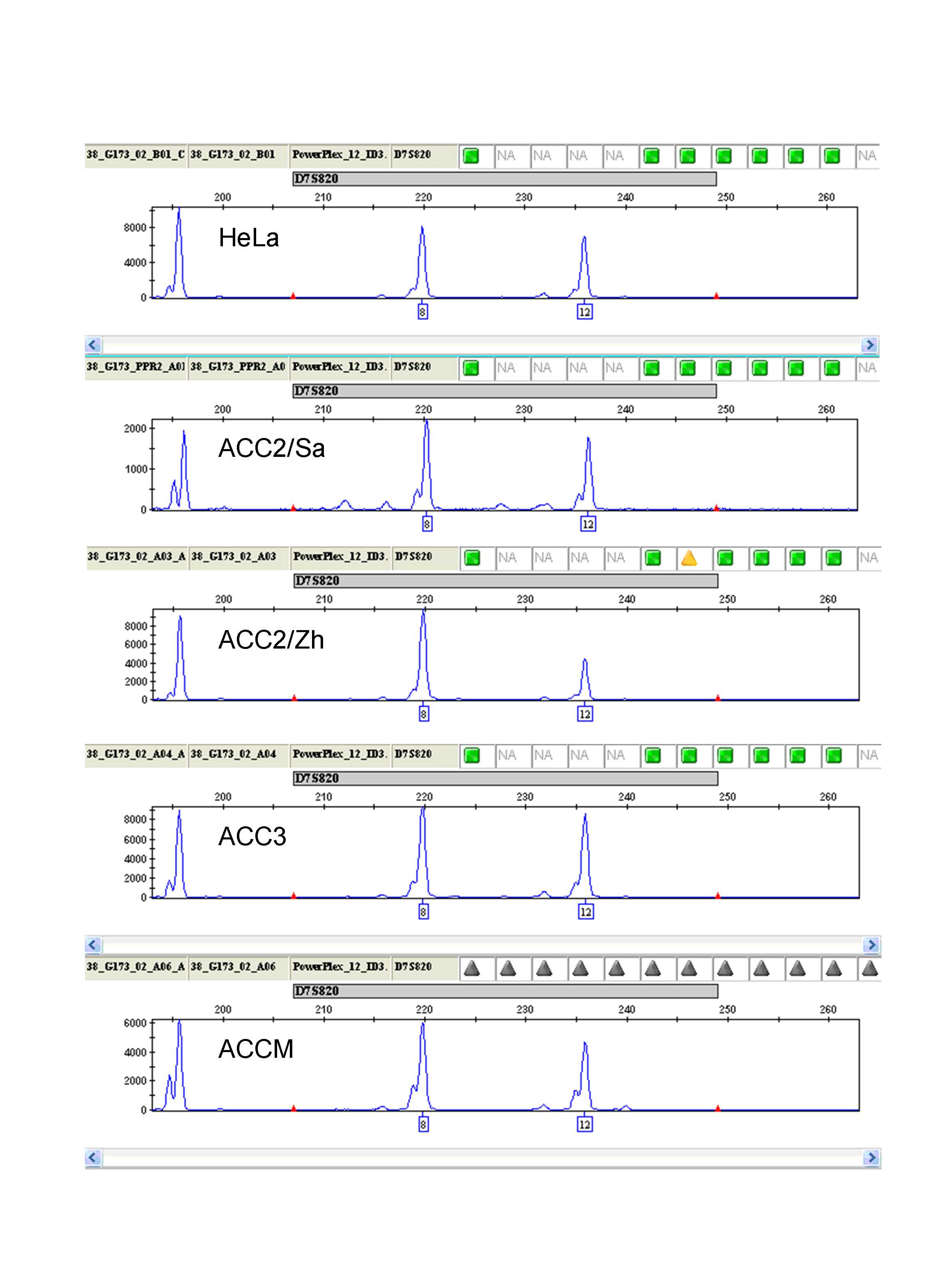

Supplement: Figure S7 — Electrophoretic profiles of the D7S820 marker for HeLa, ACC2/Sa, ACC2/Zh, ACC3, and ACCM cells shown in Table 1 are presented. (1.75 MB TIF) [file pone.0006040.s008.tif]

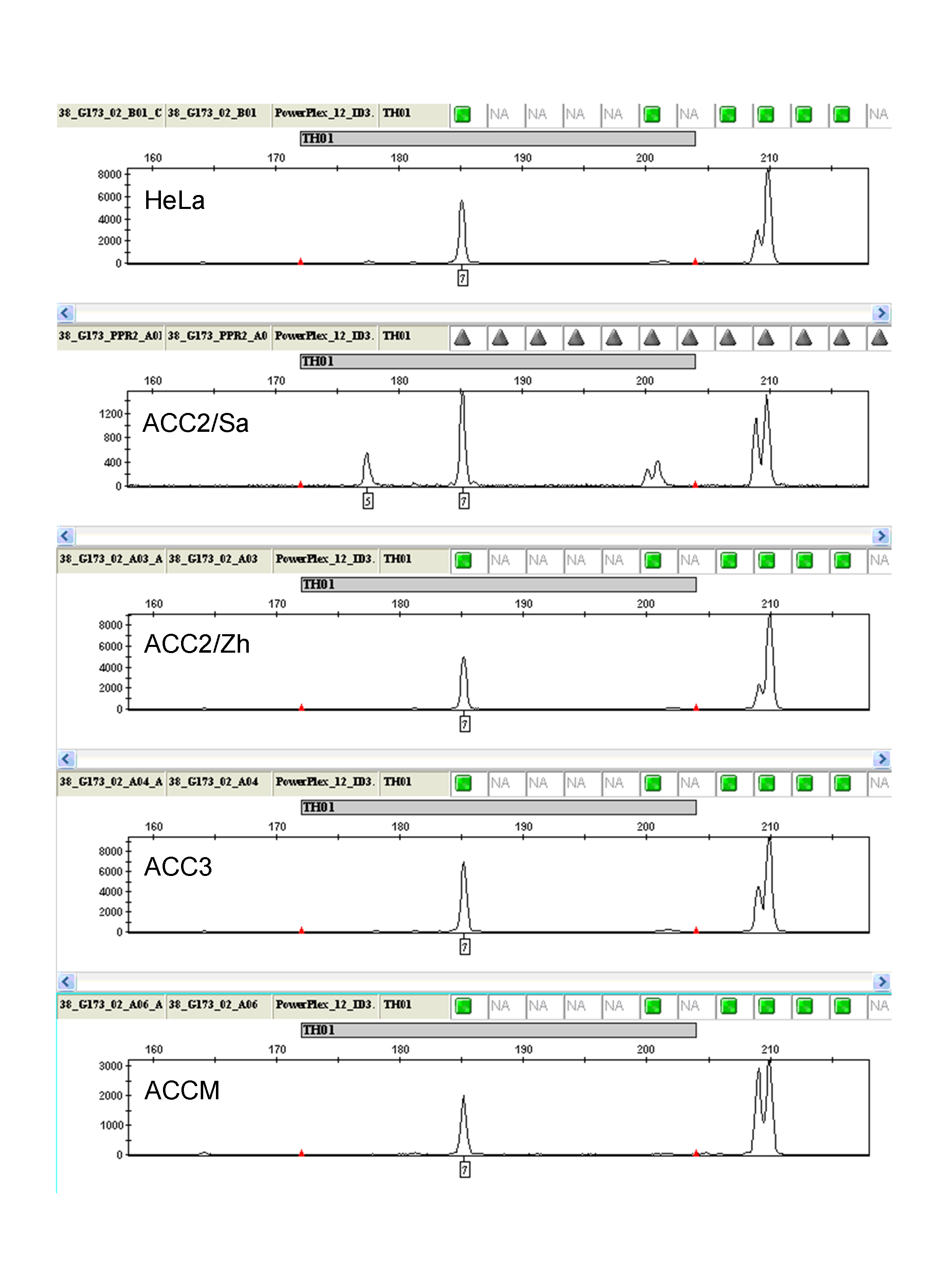

Supplement: Figure S8 — Electrophoretic profiles of the TH01 marker for HeLa, ACC2/Sa, ACC2/Zh, ACC3, and ACCM cells shown in Table 1 are presented. (1.82 MB TIF) [file pone.0006040.s009.tif]

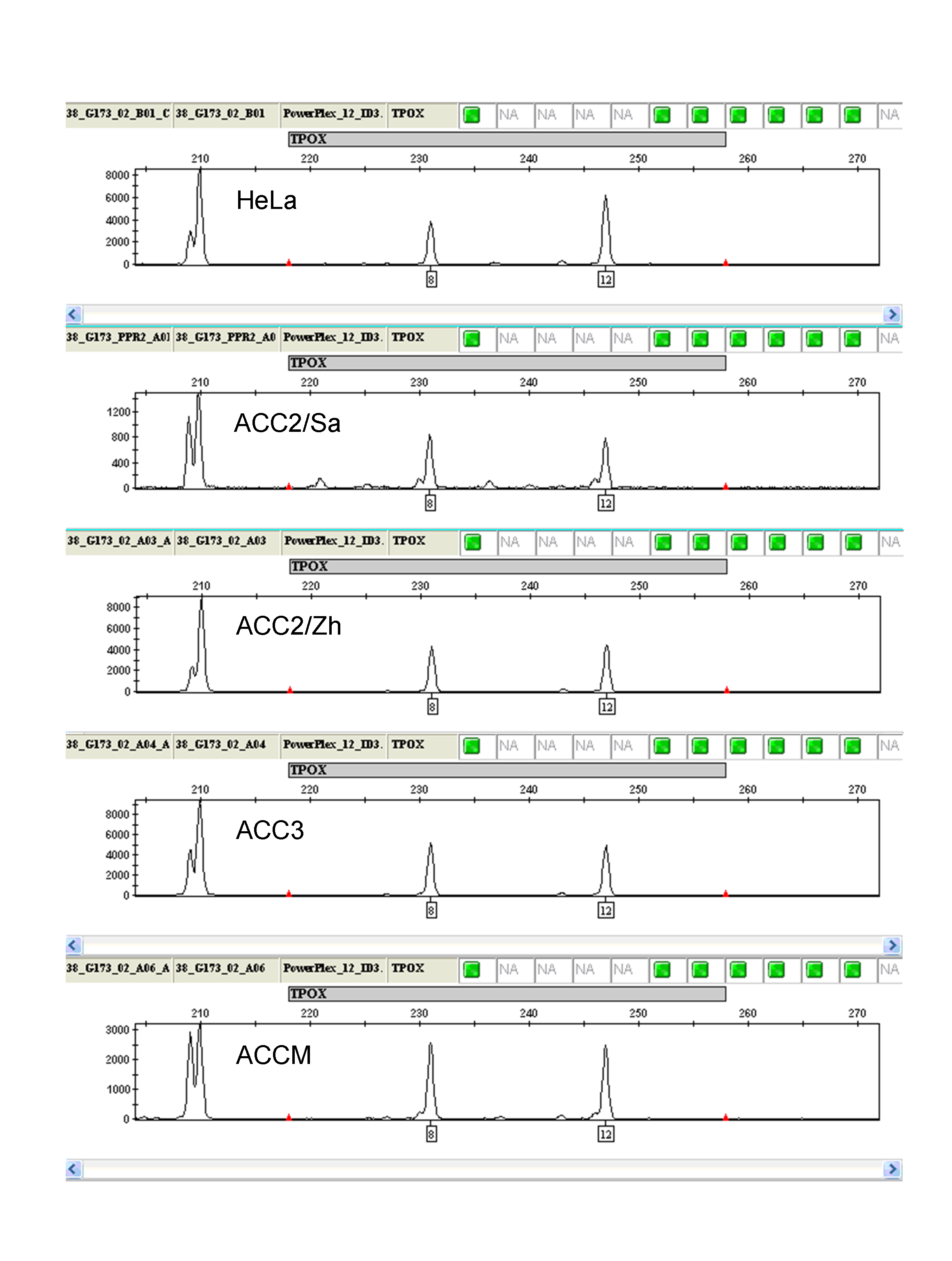

Supplement: Figure S9 — Electrophoretic profiles of the TPOX marker for HeLa, ACC2/Sa, ACC2/Zh, ACC3, and ACCM cells shown in Table 1 are presented. (1.87 MB TIF) [file pone.0006040.s010.tif]

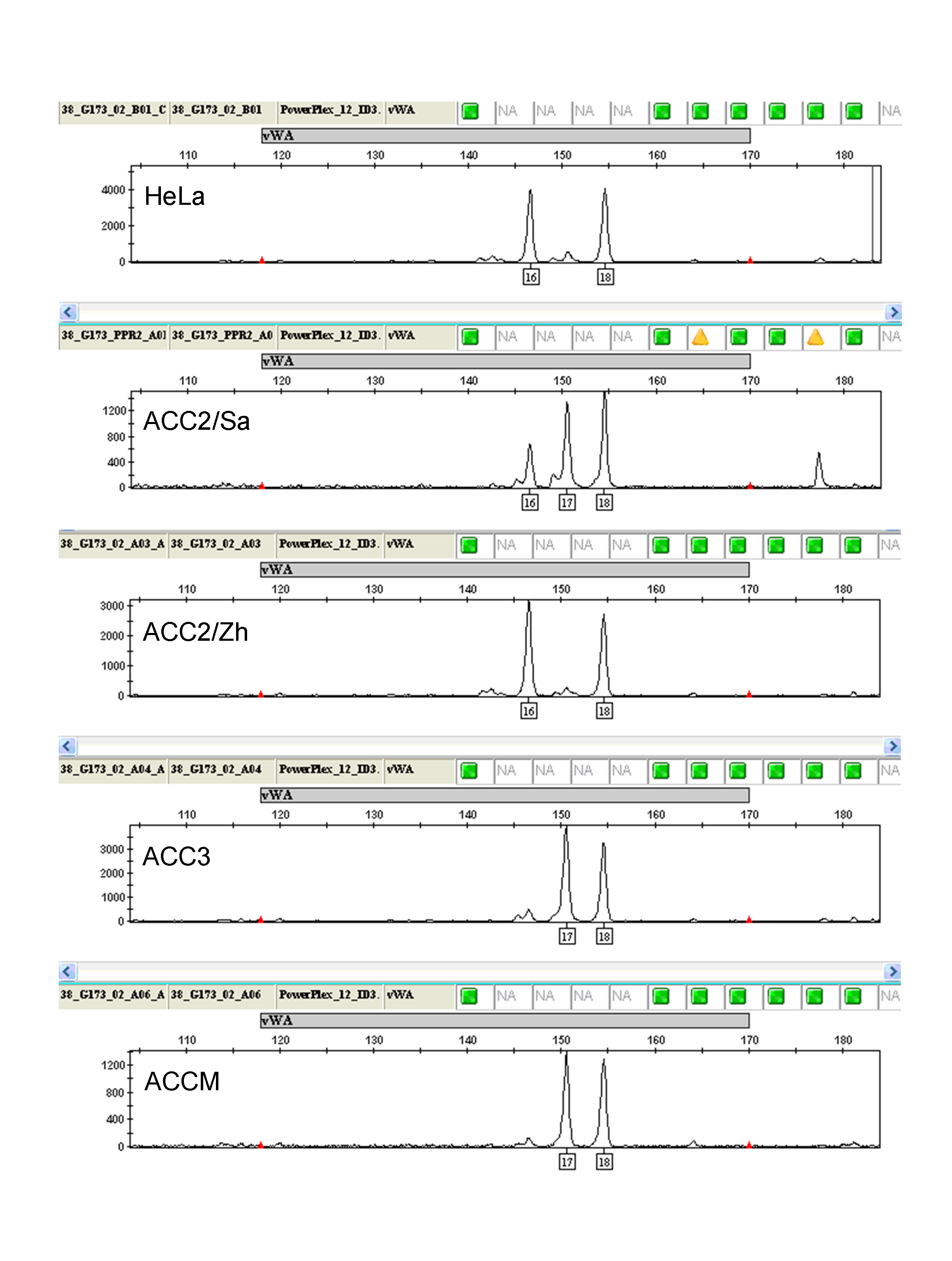

Supplement: Figure S10 — Electrophoretic profiles of the vWA marker for HeLa, ACC2/Sa, ACC2/Zh, ACC3, and ACCM cells shown in Table 1 are presented. (1.88 MB TIF) [file pone.0006040.s011.tif]

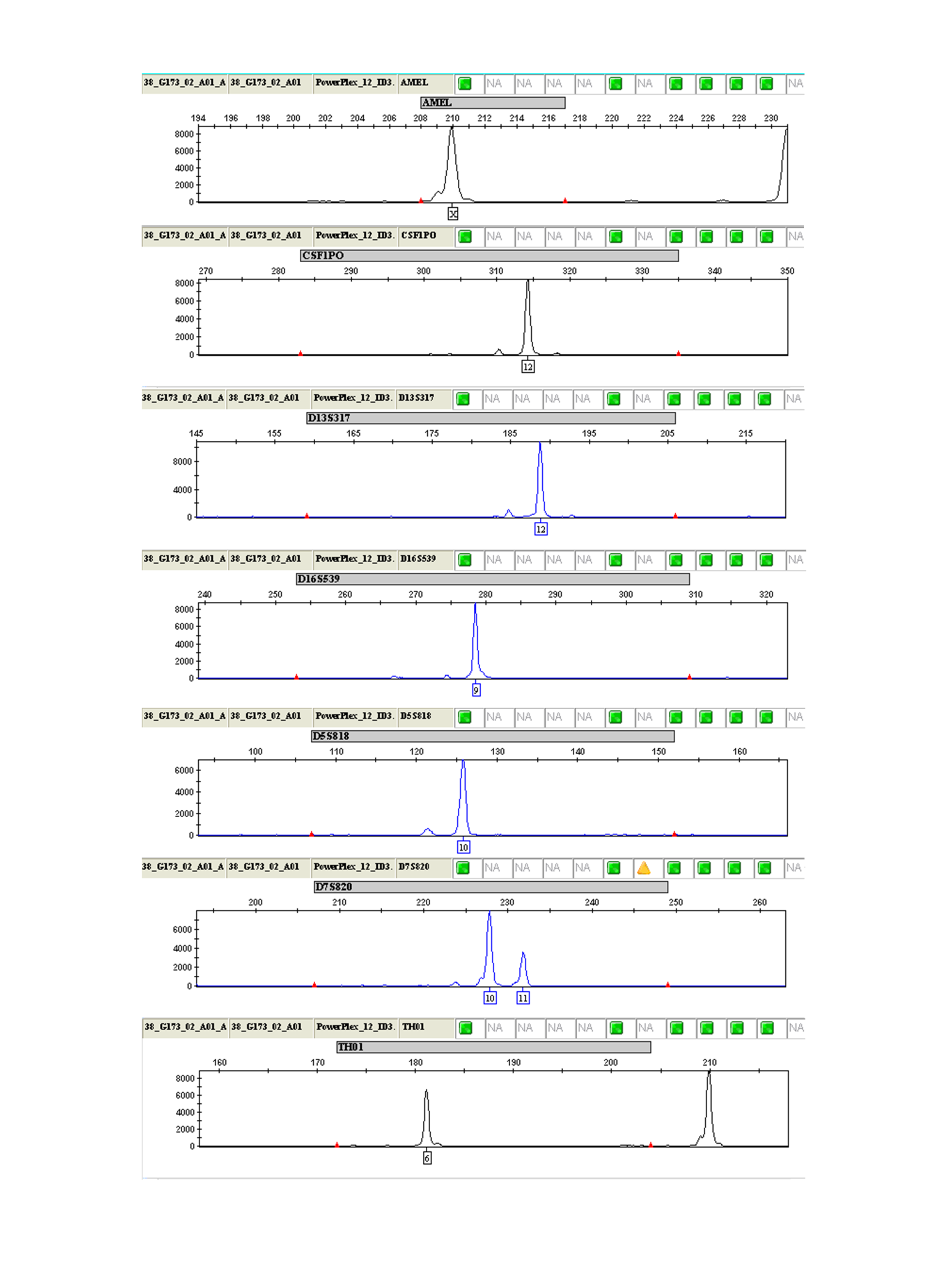

Supplement: Figure S11 — Electrophoretic profiles of the AMEL, CSF1PO, D13S317, D16S539, D5S818, D7S820, and TH01markers for ACCS cells shown in Table 2 are presented. (1.62 MB TIF) [file pone.0006040.s012.tif]

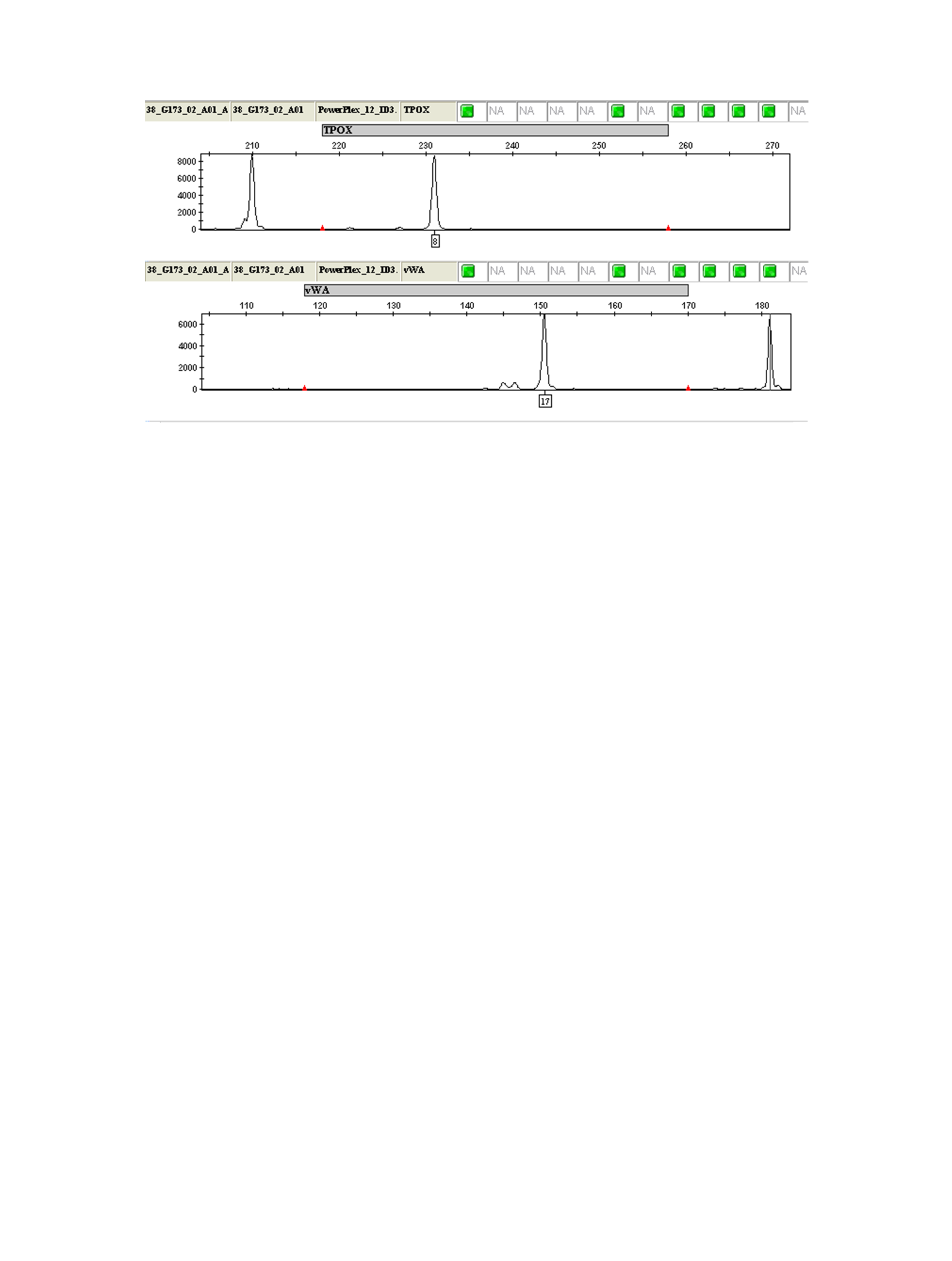

Supplement: Figure S12 — Electrophoretic profiles of the TPOX and vWA markers for ACCS cells shown in Table 2 are presented. (0.83 MB TIF) [file pone.0006040.s013.tif]
